# Differential Regulation of Ruminal Microbial Community Structure and Functional Pathways in Sheep Supplemented with Linseed Oil or Sunflower Oil

**DOI:** 10.3390/ani16111581

**Published:** 2026-05-22

**Authors:** Lu Shao, Jiaxun Dong, Ziang Wang, Peidi Zhao, Xiangpeng Yue, Wanhong Li

**Affiliations:** State Key Laboratory of Herbage Improvement and Grassland Agro-Ecosystems, College of Pastoral Agriculture Science and Technology, Lanzhou University, Lanzhou 730020, China; shaol2024@lzu.edu.cn (L.S.); dongjx2024@lzu.edu.cn (J.D.); wangza2024@lzu.edu.cn (Z.W.); zhaopd2023@lzu.edu.cn (P.Z.); lexp@lzu.edu.cn (X.Y.)

**Keywords:** Hu sheep, linseed oil, sunflower oil, serum biochemical indices, ruminal fermentation, ruminal microbiota

## Abstract

This study evaluated the effects of dietary PUFA supplementation on growth performance, serum biochemical indices, rumen morphology, ruminal fermentation, and rumen microbial communities in Hu sheep. Thirty healthy male Hu sheep (80 days old; 18.70 ± 0.72 kg) were randomly assigned to three groups (n = 10/group) and fed a basal diet supplemented with 4% rumen-bypass palmitic acid fat powder (POS), 4% linseed oil (LO; rich in ω-3 PUFA), or 4% sunflower oil (SO; rich in ω-6 PUFA). PUFA supplementation did not affect average daily gain or rumen tissue morphology (*p* > 0.05), but it significantly reduced serum creatinine, uric acid, and high-density lipoprotein concentrations (*p* < 0.05). Orthogonal comparative analysis showed that supplementing with PUFA significantly reduced acetate and propionate, while increasing isobutyrate, butyrate, isovalerate, and TVFAs (*p* < 0.05). The levels of propionate, butyrate and TVFAs in the SO group were higher than those in the LO group (*p* < 0.05), and the ratio of acetate to propionate was lower (*p* < 0.05). Supplementing with PUFA reshaped the rumen microbiota, increasing the relative abundances of Bacteroidota, Firmicutes, Euryarchaeota, Cyanobacteria, and Actinobacteriota, while decreasing Proteobacteria and Desulfobacterota (*p* < 0.05). At the genus level, *Prevotella* and *Rikenellaceae*_RC9_gut_group were enriched in both groups with added PUFA, while *Prevotellace*-UCG-001 was specifically enriched in the LO group; in contrast, *Prevotella*_7, *Succinivibriaceae*_UCG-001, *Prevotella*_9, and *Dialist* all showed a decrease (*p* < 0.05). The alpha diversity increased, while the beta diversity showed significant differences between the group with added PUFA and the control group. Functionally, LO enriches carbohydrate and energy metabolism, while SO enriches nucleotide metabolism. In summary, linseed oil and sunflower oil improved serum indices and ruminal fermentation without impairing growth, but through distinct microbial and functional pathways.

## Simple Summary

This study evaluated the effects of dietary polyunsaturated fatty acid (PUFA) supplementation on growth performance, serum biochemistry, ruminal fermentation, and microbial communities in Hu sheep. Thirty male Hu sheep (n = 10/group) were assigned to control (4% rumen-bypass palmitic acid), linseed oil (LO, ω-3), or sunflower oil (SO, ω-6) groups. PUFA supplementation did not affect growth performance or rumen morphology, but reduced serum creatinine, uric acid, and high-density lipoprotein. Orthogonal comparative analysis showed that the addition of PUFA reduced acetate and propionate, while increasing butyrate, isovalerate, and total volatile fatty acids (TVFAs); the SO group showed higher propionate, butyrate, and TVFAs but a lower A/P ratio than the LO group. PUFA reshaped the rumen microbiota, increasing Bacteroidetes and Firmicutes, decreasing Proteobacteria and Desulfobacterota, and altering multiple genera. Alpha diversity increases, while beta diversity separates the PUFA group from the control group. Functionally, LO enriches carbohydrate and energy metabolism, while SO enriches nucleotide metabolism. In summary, Linseed Oil and sunflower oil improve serum indicators and rumen fermentation through different microbial and functional pathways without harming growth.

## 1. Introduction

ω-3 and ω-6 PUFAs are essential fatty acids that play critical roles in maintaining cell membrane integrity, regulating immune function, and supporting animal productivity. Because animals cannot synthesize these fatty acids de novo, they must be supplied exogenously, most commonly through PUFA-rich plant oils in the diet [1,2,3]. Common lipid sources, including soybean oil, linseed oil, and sunflower oil, have therefore been widely used in studies of nutritional regulation in ruminants [4,5]. Unlike monogastric animals, ruminants must first expose dietary PUFA to the rumen, a specialized fermentative chamber, before intestinal absorption can occur. Most PUFAs entering the rumen undergo microbial biohydrogenation to saturated fatty acids, which changes both the composition and the amount of fatty acids reaching the small intestine [6]. In addition, excessive PUFAs can exert toxic effects on rumen microorganisms, particularly fibrolytic populations, thereby reducing fiber digestibility and altering the abundance and composition of the rumen microbiota [7]. Previous studies have mainly focused on ruminal fermentation characteristics, milk quality, and tissue fatty acid deposition [8,9]; relatively little is known about how specific PUFA sources influence the relationship between VFA production and rumen microbial community structure.

Linseed oil and sunflower oil are both rich in PUFAs, but their fatty acid profiles differ markedly. Linseed oil is enriched in α-linolenic acid (C18:3 ω-3), an ω-3 PUFA that has been associated with reduced oxidative stress, improved hepatic lipid metabolism, and enhanced antioxidant enzyme activity; lignans in flaxseed-derived products may also contribute to hepatoprotection [10]. By contrast, sunflower oil is rich in linoleic acid (C18:2 ω-6), an ω-6 PUFA that may influence hepatocyte susceptibility to apoptosis through regulation of caspase-9 and caspase-3 signaling pathways [11]. The differential effects of ω-3 and ω-6 PUFAs on rumen microorganisms may be attributed to their distinct chemical structures and biological activities. ω-3 PUFAs, particularly α-linolenic acid (C18:3), possess a higher degree of unsaturation and have been shown to exert stronger antibacterial effects against certain Gram-positive bacteria, including cellulose-digesting bacteria, thereby potentially altering the competitive dynamics within the microbial community [7,12]. In contrast, ω-6 PUFAs, such as linoleic acid (C18:2), are more readily incorporated into bacterial membranes and can modulate membrane fluidity and function [6,13]. Moreover, the biohydrogenation intermediates produced from ω-3 and ω-6 PUFAs differ, with ω-3 PUFA generating distinct conjugated linolenic acid isomers that may selectively inhibit or promote specific bacterial taxa [13,14]. These differences collectively shape the ruminal microbial ecosystem and its metabolic outputs. Given these structural and functional differences, we hypothesized that dietary supplementation with linseed oil (ω-3) and sunflower oil (ω-6) would differentially alter ruminal microbial community structure and metabolic pathways, leading to distinct volatile fatty acid profiles. Indeed, altering the dietary ω-6:ω-3 fatty acid ratio has been shown to change ruminal fermentation parameters and microbial populations in goats [14]. However, it remains unclear how ω-3- and ω-6-rich oils drive distinct fermentation patterns through remodeling of the rumen microbiota. Clarifying these mechanisms is important for the precise nutritional regulation of rumen fermentation.

Hu sheep are one of the most widely raised mutton sheep breeds in China because of their early sexual maturity and high reproductive performance. The present study was designed to elucidate how rumen microorganisms respond to different dietary PUFA sources. To this end, a basal diet was supplemented with 4% rumen-bypass palmitic acid fat powder (as a saturated fatty acid source), 4% linseed oil (as an ω-3 PUFA source), or 4% sunflower oil (as an ω-6 PUFA source). We evaluated growth performance, serum biochemical indices, rumen tissue morphology, and ruminal VFAs. Furthermore, 16S rRNA high-throughput sequencing was used to characterize changes in the rumen microbial community, and PICRUSt2 was employed to predict potential functional pathways through which different PUFA sources may regulate rumen fermentation.

## 2. Materials and Methods

### 2.1. Experimental Design, Animals, Diet and Management

The feeding trial was conducted at Defu Agricultural Technology Co., Ltd. (Wuwei, China). All animal procedures were approved by the Animal Care and Use Committee of Lanzhou University (CY20230913). Thirty healthy male Hu lambs aged 80 days (18.70 ± 0.72 kg) with similar body weights were randomly assigned to three treatment groups (n = 10 per group): a palmitic acid group (POS), a linseed oil group (LO), and a sunflower oil group (SO). The three groups were fed a basal diet supplemented with 4% rumen-bypass palmitic acid fat powder, 4% linseed oil, or 4% sunflower oil, respectively. Diets were formulated according to the Nutritional Requirements for Meat Sheep (NY/T 816-2021) and prepared as total mixed pellet feeds; detailed ingredient and nutrient compositions are provided in Table 1. The fatty acid composition of each diet was determined by gas chromatography and is summarized in Table 2. All experimental diets were supplied by Gansu Runmu Biological Engineering Co., Ltd. (Jinchang, Gansu, China). Immunization and disinfection procedures in the sheep barn were conducted according to the farm’s routine management protocol. The pretrial period lasted 7 days, during which the basal diet was gradually replaced by the experimental diets. The formal trial lasted 84 days. Sheep were fed twice daily at 08:00 and 18:00, and feed and water were provided ad libitum throughout the experiment.

### 2.2. Sampling and Analysis

For all phenotypic and omics measurements, samples were collected from all 10 sheep in each treatment group, and the data were averaged within each group for subsequent statistical analysis.

#### 2.2.1. Production Performance

Initial body weight (IBW) and final body weight (FBW) were recorded before the morning feeding on days 1 and 84 using a bench scale. Average daily gain (ADG) was calculated as (FBW − IBW) divided by the number of experimental days.

#### 2.2.2. Blood Sampling and Analysis

At the end of the trial period, after a 12 h fasting period (from 20:00 the previous evening to 08:00 the next morning) with free access to water, blood samples were collected from the jugular vein at 08:00, before the morning feeding. After standing for 2–3 h, samples were centrifuged at 3000 rpm for 10 min to obtain serum, which was stored at −80 °C until analysis. Serum biochemical analysis was conducted at the Beijing Sinouk Institute of Biological Technology (Beijing, China) using a Mindray BS-420 automatic biochemical analyzer (Shenzhen Mindray Bio-Medical Electronics Co., Ltd., Shenzhen, China). The measured indices included lipid-related parameters (total cholesterol, triglycerides, high-density lipoprotein, and low-density lipoprotein), renal function indicators (creatinine, urea, and uric acid), and liver function indicators (alkaline phosphatase, aspartate aminotransferase, alanine aminotransferase, and total bilirubin).

#### 2.2.3. Collection and Analysis of Rumen VFA

Immediately after slaughter, the abdominal cavity was opened and approximately 50 mL of rumen contents was collected from three sites in the rumen (the anterior dorsal sac, posterior dorsal sac, and ventral sac) [15]. Equal volumes from the three sites were mixed and filtered through four layers of cotton gauze. A 5 mL aliquot of the filtrate was immediately stored at −80 °C for rumen microbiota analysis. An additional 10 mL aliquot was mixed with 25% metaphosphoric acid at a 1:5 ratio and stored at −20 °C for VFA analysis. Ruminal VFA concentrations were determined by gas chromatography (Thermo Scientific TRACE 1300, Milan, Italy) using a DB-FFAP capillary column (30 m × 0.32 mm × 0.25 μm, Agilent, Santa Clara, CA, USA), with an injection volume of 1 μL and a split ratio of 50:1 [16].

#### 2.2.4. Rumen Tissue Morphology Analysis

A tissue sample (approximately 1 cm^2^) was collected from the ventral sac of the rumen, gently rinsed with pre-cooled physiological saline to remove adherent digesta, and fixed in Sevier universal tissue fixative (Sevier Biotechnology Co., Ltd., Wuhan, China) for 48 h. Hematoxylin and eosin (H&E) staining and section preparation were performed by Wuhan Sevier Biotechnology Co., Ltd. Whole-slide images were obtained using an Olympus VS120 digital slide scanner (Olympus, Japan). Morphological measurements were performed with Olympus cellSens software (V4.3), including (1) papilla length and width and (2) the thickness of each epithelial layer in the non-papillary mucosal area: the stratum corneum (from the epithelial surface to the beginning of the granular layer), the stratum granulosum (from the lower edge of the stratum corneum to the beginning of the stratum spinosum), the combined stratum spinosum and basal layer (from the lower edge of the granular layer to the lamina propria), and total epithelial thickness. Epithelial layers were defined according to Graham and Simmons [17].

#### 2.2.5. Analysis of Rumen Microbiota

Rumen fluid samples were snap-frozen in liquid nitrogen and sent to Novogene Biotechnology Co., Ltd. (Beijing, China) for 16S rRNA gene sequencing. Microbial DNA was extracted from rumen fluid using a modified CTAB method (Nobleryder, Beijing, China). After lysozyme lysis, phenol–chloroform–isoamyl alcohol extraction, isopropanol precipitation, 75% ethanol washing, and RNase A digestion, purified DNA was obtained. The V4 region of the bacterial 16S rRNA gene was amplified using primers 515F–806R (forward: GTGYCAGCGMGCGCGGTAA; reverse: GGACTACNNGGTATCTAAT). Each PCR reaction contained 15 μL Phusion High-Fidelity PCR Master Mix (New England Biolabs, Ipswich, MA, USA), 0.2 μM of each primer, and 10 ng genomic DNA template. Amplification was performed in a Bio-Rad gradient PCR instrument under the following conditions: initial denaturation at 98 °C for 1 min; 30 cycles of 98 °C for 10 s, 50 °C for 30 s, and 72 °C for 30 s; and a final extension at 72 °C for 5 min. Amplicons were purified with magnetic beads, and libraries were constructed and quantified using a Qubit 2.0 fluorometer (Thermo Fisher Scientific, Waltham, MA, USA). After library quality verification, sequencing was performed on an Illumina NovaSeq 6000 platform (Illumina, San Diego, CA, USA) in PE250 mode.

After barcode and primer trimming, paired-end reads were merged using FLASH (V1.2.11) to generate raw tags. Raw tags were then quality-filtered with fastp (V0.23.1) to obtain clean tags, which were aligned against the Silva and Unite reference databases to remove chimeric sequences and generate effective tags. Noise reduction was performed using either the DADA2 module or the deblur function in QIIME2 (Version 2022.2), yielding the final amplicon sequence variants (ASVs) and feature table. Taxonomic annotation was performed in QIIME2 using the Silva138.1 database for 16S and 18S sequences and Unite v9.0 for ITS sequences; non-canonical regions were annotated against the micro_NT database by default. Alpha and beta diversity were subsequently calculated in QIIME2 (Version 2022.2). Bray–Curtis distances were used to evaluate microbial community structure, and non-metric multidimensional scaling (NMDS) and principal coordinate analysis (PCoA) were performed in R (V4.0.3) using the ade4 (V4.0.3) and ggplot2 (V4.0.3) packages. ANOSIM and ADONIS (PERMANOVA) from the vegan package (v2.6-4) in R (v4.0.3) were used to test differences among groups. Relative abundances of the top 10 phyla and genera were analyzed by one-way analysis of variance. PICRUSt2 (2.3.0) was used to predict functional metabolic pathways of the rumen microbiota. Based on the ASV table, the default PICRUSt2 database was used to estimate the relative abundance of the top 20 Meta Cyc pathways in each sample and to identify differentially enriched pathways. LEfSe (V1.1.01) was used to analyze differentially enriched functional pathways and generate LDA score plots.

### 2.3. Statistical Analysis

Data were analyzed using SPSS 25.0 (IBM Corp., Armonk, NY, USA) and presented as means ± standard error of the mean (SEM). Orthogonal contrast analysis was used as the primary statistical method to decompose the treatment effects into two pre-planned comparisons: Contrast 1 compared the control group (POS) with the two PUFA-supplemented groups (LO and SO combined) to assess the overall effect of PUFA supplementation; Contrast 2 compared the LO group with the SO group to evaluate the differential effects between ω-3 and ω-6 PUFA sources. Differences were considered significant at *p* < 0.05.

## 3. Results

### 3.1. Effects of Linseed Oil and Sunflower Oil on the Growth Performance of Hu Sheep

Orthogonal contrast analysis showed no significant effects of PUFA supplementation on any growth performance parameter (Table 3; Contrast 1 and Contrast 2, all *p* > 0.05).

### 3.2. Effects of Linseed Oil and Sunflower Oil on Blood Biochemical Parameters in Hu Sheep

As shown in Table 4, orthogonal contrast analysis revealed that PUFA supplementation (Contrast 1: POS vs. LO + SO) significantly decreased serum CREA (*p* < 0.05), UA (*p* < 0.05), AST (*p* < 0.05), and HDL (*p* < 0.05). No significant effects were observed for any other serum biochemical parameters (all *p* > 0.05).

### 3.3. Effects of Linseed Oil and Sunflower Oil on Rumen Tissue Morphology in Hu Sheep

**Figure 1:**
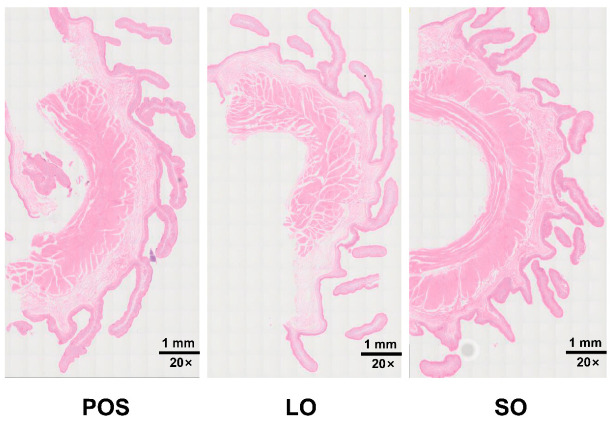
Histological morphology of the ventral rumen sac in Hu sheep fed palm-based saturated fat (POS), linseed oil (LO), or sunflower oil (SO). Scale bar = 1 mm.

As shown in Table 5, orthogonal comparative analysis shows that Contrast 1: POS and LO + SO, all *p* > 0.05, and there was no significant difference between the LO and SO groups. Adding 4% linseed oil or sunflower oil to the diet had no effect on the rumen tissue morphology of Hu sheep. H&E staining (Figure 1) further showed that rumen tissue structure remained intact in all three groups, with no obvious pathological lesions.

### 3.4. Effects of Linseed Oil and Sunflower Oil on Ruminal VFA Profiles in Hu Sheep

As shown in Table 6, orthogonal comparative analysis showed that supplementing with PUFAs (Contrast 1: POS and LO + SO) significantly reduced acetate and propionate, while increasing isobutyrate, butyrate, isovalerate, and TVFAs. In addition, the content of propionate, butyrate, and TVFAs in the SO group was higher than that in the LO group, and the A/P ratio was lower than that in the LO group (contrast 2: LO and SO).

### 3.5. Analysis of the Rumen Microbial Community

#### 3.5.1. Effects of Linseed Oil and Sunflower Oil on Rumen Microbial Community Structure in Hu Sheep

**Figure 2:**
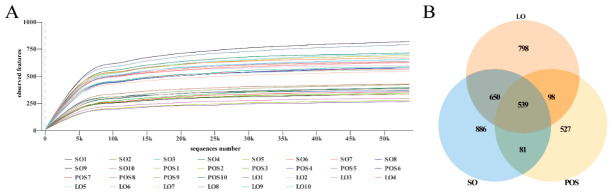
Diversity and community composition of the rumen microbiota. (**A**) Rarefaction curves. (**B**) Venn diagram of shared and unique ASVs.

The rarefaction curves for all groups reached a plateau, indicating that sequencing depth was sufficient to capture the major features of the rumen microbiota (Figure 2A). In total, 5486 ASVs were identified across all samples, of which 539 (9.83%) were shared among the three groups. The POS, LO, and SO groups contained 1245, 2085, and 2156 ASVs, respectively, with 527, 798, and 886 unique ASVs (Figure 2B). At the phylum level (Table 7), Bacteroidota and Firmicutes were dominant across all groups, together accounting for more than 80% of the total relative abundance. Orthogonal contrast analysis revealed that PUFA supplementation (Contrast 1) significantly increased the relative abundances of Bacteroidota, Firmicutes, Euryarchaeota, Cyanobacteria, and Actinobacteriota (*p* < 0.001), while significantly decreasing Proteobacteria (*p* = 0.001) and Desulfobacterota (*p* < 0.001). Spirochaetota showed no significant change (*p* = 0.058). For Contrast 2 (LO vs. SO), only Desulfobacterota differed significantly between the two PUFA sources (*p* < 0.001), with no other phyla showing significant differences (*p* > 0.05). At the genus level (Table 8), *Prevotella*_7 was dominant in the POS group, whereas *Prevotella* was dominant in both the LO and SO groups. Relative abundances of *Prevotella*_7, *Succinivibrionaceae*_UCG-001, *Prevotella*_9, and *Dialister* were significantly lower in the LO and SO groups than in the POS group (*p* < 0.05), whereas *Prevotella* and *Rikenellaceae*_RC9_gut_group were significantly higher. *Prevotellaceae*_UCG-001 was specifically enriched in the LO group relative to both the POS and SO groups (*p* < 0.05).

#### 3.5.2. Alpha-Diversity Analysis

Alpha-diversity indices are presented in Table 9. Compared with the POS group, the LO and SO groups showed significantly higher Chao1, Pielou’s evenness, Shannon index, and observed features (*p* < 0.05), whereas Simpson’s index did not differ significantly among groups (*p* > 0.05).

#### 3.5.3. Beta-Diversity Analysis

**Figure 3:**
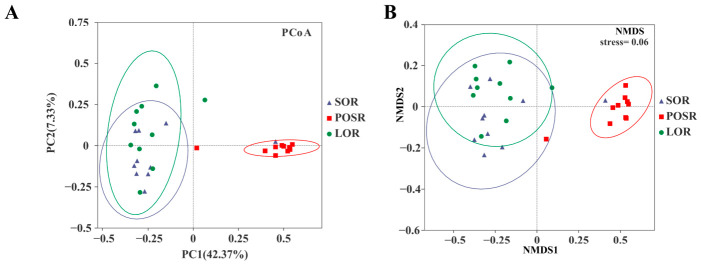
Colors and symbols for different groups correspond to those in the legend. Principal coordinate analysis (PCoA) (**A**) and non-metric multidimensional scaling (NMDS) (**B**) of rumen microbial communities.

As shown in Figure 3A,B, rumen microbial community structure differed markedly between the POS group and the two PUFA-supplemented groups. Both PCoA and NMDS showed clear separation of the three treatment clusters. PERMANOVA indicated significant differences between POS and both LO and SO, whereas the LO and SO groups were not significantly separated. ANOSIM produced the same overall pattern (Table 10).

#### 3.5.4. Differential Taxa and Functional Prediction Analysis

**Figure 4:**
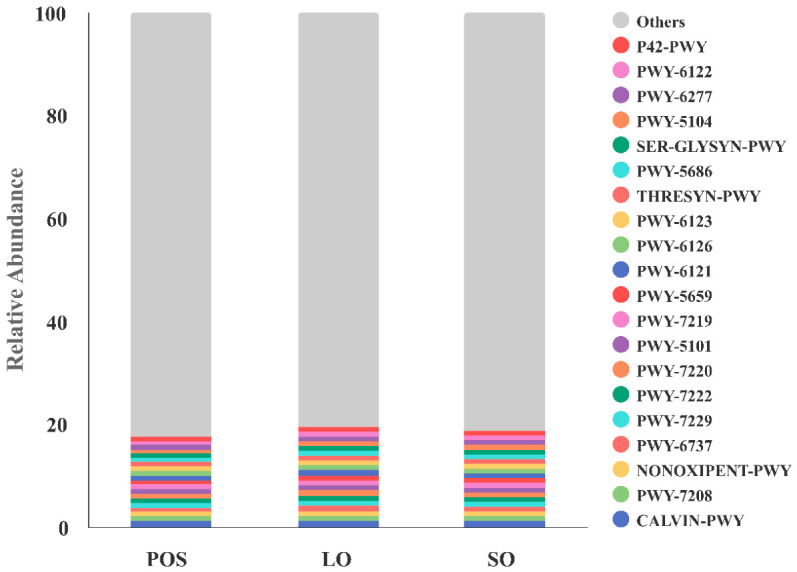
Top 20 predicted functional pathways of the rumen microbiota.

**Figure 5:**
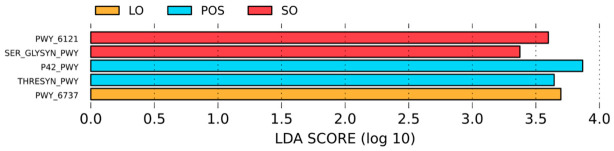
Differentially enriched metabolic pathways predicted for the rumen microbiota. Note: The English names of the metabolic pathways discussed in this study are listed below. According to the annotations on the right side of Figure 5, from bottom to top, they are: CALVIN-PWY, Calvin–Benson–Bassham cycle; PWY-7208, superpathway of pyrimidine nucleobase salvage; NONOXIPENT-PWY, pentose phosphate pathway (non-oxidative branch); PWY-7229, superpathway of adenosine nucleotides de novo biosynthesis I; PWY-7219, de novo biosynthesis of adenosine ribonucleotides; PWY-5101, L-isoleucine biosynthesis II; PWY-6123, inosine-5’-phosphate biosynthesis I; PWY-6126, superpathway of adenosine nucleotides de novo biosynthesis II; PWY-7222, de novo biosynthesis of guanosine deoxyribonucleotides II; PWY-7220, de novo biosynthesis of adenosine deoxyribonucleotides II; PWY-6737, starch degradation V; PWY-6121, 5-aminoimidazole ribonucleotide biosynthesis I; THRESYN-PWY, superpathway of L-threonine biosynthesis; P42-PWY, incomplete reductive TCA cycle; PWY-5659, GDP-mannose biosynthesis; SER-GLYSYN-PWY, superpathway of L-serine and glycine biosynthesis I; PWY-5104, L-isoleucine biosynthesis IV; PWY-5686, UMP biosynthesis; PWY-6277, superpathway of 5-aminoimidazole ribonucleotide biosynthesis; PWY-6122, 5-aminoimidazole ribonucleotide biosynthesis II.

Functional prediction analysis (Figure 4 and Figure 5) showed that the rumen microbiota of the three groups differed in metabolic pathway abundance. The top 20 predicted pathways were mainly related to nucleotide metabolism, amino acid biosynthesis, carbohydrate metabolism, and energy metabolism. Relative to the other groups, the POS group was characterized primarily by pathways involved in nucleotide metabolism and amino acid biosynthesis, including the incomplete reductive tricarboxylic acid cycle (P42-PWY) and the superpathway of L-threonine biosynthesis (THRESYN-PWY). The LO group was mainly enriched in carbohydrate- and energy-metabolism pathways, particularly the non-oxidative pentose phosphate pathway (NONOXIPENT-PWY) and starch degradation V (PWY-6737). The SO group was predominantly enriched in nucleotide-metabolism pathways, especially 5-aminoimidazole ribonucleotide biosynthesis I (PWY-6121).

## 4. Discussion

In the present study, dietary supplementation with linseed oil or sunflower oil did not affect growth performance or rumen tissue morphology in Hu sheep, consistent with previous reports [18,19,20,21,22]. These results suggest that moderate oil supplementation does not necessarily compromise growth performance or rumen epithelial structure in ruminants. Serum biochemical indices revealed that PUFA supplementation significantly reduced creatinine, uric acid, and HDL, with AST also lower in the SO group, suggesting potential benefits for renal and hepatic metabolic status. AST is widely used as an indicator of liver cell injury because it is released into the circulation when hepatocellular membrane integrity is compromised [23].

The changes in serum biochemical indicators are closely related to rumen fermentation, with the core being that VFAs produced by rumen microbial fermentation are absorbed by the rumen epithelium and enter the portal venous circulation, and are then taken up and metabolized by the liver. This process directly regulates the levels of multiple serum biochemical parameters. Specifically, propionate is the major precursor for gluconeogenesis [24,25], acetate supports peripheral energy metabolism, and butyrate may indirectly reduce hepatic burden by improving intestinal barrier function and epithelial health [26]. For this reason, changes in rumen fermentation and microbial ecology provide important mechanistic clues for interpreting the serum responses observed here. Orthogonal contrast analysis showed that PUFA supplementation decreased acetate and propionate and increased isobutyrate, butyrate, isovalerate, and TVFAs, consistent with previous reports. Dietary unsaturated fatty acids selectively inhibit rumen microbes, reshaping microbial metabolism [12,13]. For example, 6% sunflower or linseed oil increased propionate and decreased butyrate and the A/P ratio, and different oils altered butyrate and propionate depending on unsaturation [27,28]. In our study, the concurrent rise in TVFAs and microbial diversity suggests enhanced fermentation, likely due to enrichment of polysaccharide-degrading phylum like Bacteroidota and Firmicutes. The SO (ω-6) group had higher butyrate, TVFAs, and propionate, and a lower A/P ratio than the LO (ω-3) group. Consistent with previous reports, different oil sources can differentially influence ruminal fermentation characteristics [29]. This variation is attributed to structural differences: α-linolenic acid (C18:3, ω-3) has stronger antibacterial effects against hydrogenating bacteria than linoleic acid (C18:2, ω-6) [12], so stronger selection in LO may suppress propionate- and butyrate-related microbes. Supporting this, lowering the ω-6:ω-3 ratio in goats reduced propionate [14], a trend partially consistent with our findings.

PUFA supplementation significantly increased alpha-diversity indices (Chao1, observed features, Shannon, and Pielou-e), indicating enhanced microbial richness and evenness. Greater microbial diversity is generally associated with improved ecological stability, stress resistance, and host health [30,31]. Higher richness and evenness typically improve fermentation efficiency and substrate utilization, thereby increasing the production of fermentation end products [32]. Consistently, total VFA concentration was significantly elevated in both the LO and SO groups, supporting this interpretation. The PCoA and NMDS analysis clearly separated the PUFA-supplemented groups from the POS group, indicating that dietary fatty acid source substantially reshaped the rumen microbial community. Similar responses have been reported previously; for example, Sears et al. [33] showed that palmitic, stearic, and oleic acids altered rumen fiber digestibility and microbial composition, while Petri et al. [34] demonstrated that linseed and sunflower seed supplementation changed multiple ruminal bacterial genera and was closely associated with tissue fatty acid profiles.

At the phylum level, PUFA supplementation increased Bacteroidota and Firmicutes while decreasing Proteobacteria and Desulfobacterota. Bacteroidota and Firmicutes are major degraders of dietary polysaccharides and are closely linked to host energy metabolism [35]. Their increased abundance in the LO and SO groups therefore suggests enhanced fermentation of complex dietary substrates. This increase may help explain the higher TVFA concentrations observed in the PUFA-fed groups. Additionally, only Desulfobacterota showed significant differences between the two sources of PUFA, indicating that most of the component changes at the phylum level were similar between LO and SO treatments. Although the number of Spirochaetota increased in the group with PUFA added, orthogonal comparative analysis was at the edge of significance (*p* = 0.058). However, the known association between Spirochaetota and cellulose degradation and hydrogen metabolism [36,37], as well as the consistency of numerical trends between the two groups with added PUFAs, suggest a potential role worth further investigation, which can be clarified in the future through larger sample sizes or more targeted functional analysis. At the genus level, the microbial community shifted from *Prevotella*_7 dominance in the POS group to *Prevotella* dominance in the LO and SO groups. Although *Prevotella* is associated with acetate and butyrate production [38], acetate and propionate levels were unexpectedly lower in PUFA-fed groups. This apparent contradiction may be explained by the concurrent reduction in Succinivibrionaceae_UCG-001, a key succinate-producing taxon that supplies the precursor for propionate formation [27]. Notably, *Prevotellaceae*_UCG-001 was significantly enriched in the LO group, Wang et al. [39] similarly reported that the abundance of *Prevotellaceae*_UCG-001 was significantly higher in the treatment group with higher ruminal n-3 PUFA levels, while *Rikenellaceae*_RC9_gut_group was enriched in both PUFA-supplemented groups, indicating that ω-3-rich linseed oil may select for a distinct carbohydrate-utilizing bacterial consortium. *Prevotella*_7 and *Prevotella*_9 possess different carbohydrate-active enzyme repertoires [40], and their reduction may have further limited the availability of fermentation substrates for propionate formation, thereby contributing to the observed decrease in propionate levels. However, their exact metabolic roles require further investigation. In addition, the enrichment of *Rikenellaceae*_RC9_gut_group in the PUFA-supplemented groups suggests enhanced fiber degradation under lipid supplementation [41]. Biohydrogenation of unsaturated fatty acids in the rumen is mediated by specialized microbial communities and likely underlies part of the differential response to linseed and sunflower oils. Classical hydrogenating taxa, particularly members of the genus *Butyrivibrio*, are known to participate in the conversion of linoleic and linolenic acids to intermediate products and ultimately to stearic acid [42]. These unsaturated fatty acids can also inhibit biohydrogenating bacteria until detoxification proceeds, highlighting the strong selective pressure imposed by dietary lipids on the rumen ecosystem [12]. Although such classical hydrogenating bacteria were not among the top 10 genera detected in the present study, the functional prediction results provide indirect evidence that different PUFA sources induced distinct metabolic adaptations. This interpretation is consistent with broader rumen metagenomic evidence showing that carbohydrate metabolism, amino acid metabolism, and nucleotide metabolism are core microbial functions in the ruminant gastrointestinal tract [37].

Functional prediction using PICRUSt2 revealed distinct metabolic priorities for the two PUFA sources. The LO group was enriched in carbohydrate and energy metabolism pathways, whereas the SO group was mainly enriched in nucleotide metabolism pathways. Specifically, the LO group was enriched in pentose phosphate pathway-related and starch-degradation functions, suggesting enhanced carbohydrate turnover under linseed oil supplementation. Because α-linolenic acid contains three double bonds, its transformation in the rumen is generally more complex than that of linoleic acid [13,43]. By contrast, the SO group was mainly enriched in nucleotide-metabolism pathways, particularly 5-aminoimidazole ribonucleotide biosynthesis I, a key step in de novo purine synthesis. Enhanced nucleotide biosynthesis is commonly associated with increased microbial proliferation and metabolic activity [44,45]. Thus, the simpler hydrogenation demands of linoleic acid may have allowed the SO-associated microbiota to allocate more resources toward proliferation-related functions. The functional predictions suggest that these differences may be linked to distinct metabolic priorities of the rumen microbiota under ω-3- and ω-6-rich conditions, rather than to a simple uniform effect of all unsaturated oils. Thus, linseed oil and sunflower oil remodel the ruminal microbiota and fermentation via distinct metabolic priorities—carbohydrate/energy metabolism for ω-3 and nucleotide metabolism for ω-6—without impairing growth performance. Future metagenomic and metabolomic studies are warranted to verify the underlying microbial genes and pathways involved.

## 5. Conclusions

In conclusion, dietary linseed oil (ω-3 PUFA) and sunflower oil (ω-6 PUFA) improved ruminal fermentation by decreasing acetate and propionate, while increasing isobutyrate, butyrate, isovalerate, and TVFA and altered rumen microbial community structure in Hu sheep without impairing growth performance or rumen tissue morphology. Moreover, the SO group exhibited higher butyrate, TVFAs, and propionate, and a lower acetate/propionate ratio, than the LO group. Both oils increased microbial diversity and TVFA production. They were associated with distinct microbial taxa and predicted functional pathways: linseed oil preferentially enriched carbohydrate- and energy-metabolism functions, whereas sunflower oil preferentially enriched nucleotide-metabolism functions. These findings provide new insight into how different PUFA sources modulate rumen microecology and may inform the precision use of lipid supplements in ruminant nutrition.

## Figures and Tables

**Table 1:** Ingredient composition and nutrient levels of the experimental diets (DM basis, %).

Items		Treatments	
	POS	LO	SO
Ingredients %			
Corn stalk	24	24	24
Corn husk	8	8	8
Cracked corn	26	26	26
Soybean meal	5	5	5
Cottonseed meal	6	6	6
Corn germ meal	20	20	20
Rumen-fermented palm fat powder	4	0	0
Sunflower oil	0	0	4
Linseed oil	0	4	0
Urea granules	1	1	1
Molasses	3	3	3
NaCl	0.5	0.5	0.5
Limestone	1.2	1.2	1.2
Sodium bicarbonate	0.8	0.8	0.8
Premix	0.5	0.5	0.5
Total	100	100	100
Nutrient levels			
Dry matter	92.26	90.92	91.45
Crude protein	15.42	15.48	15.41
Metabolizable energy (MJ/kg)	12.57	12.64	12.57
Neutral detergent fiber	31.43	32.76	32.10
Acid detergent fiber	15.60	14.14	14.13

Note: Dry matter, crude protein, neutral detergent fiber, and acid detergent fiber were measured, whereas metabolizable energy was calculated. The premix provided vitamins and minerals. Each kilogram of diet dry matter contained 2500 IU vitamin A, 23 IU vitamin E, 0.3 mg selenium, 70 mg iron, 41 mg zinc, and 8 mg copper.

**Table 2:** Dietary fatty acid composition (%).

Items		Treatments	
	POS	LO	SO
C16:0 Palmitic acid	49.55	12.03	10.80
C18:2n6t Linoleic acid	26.10	32.34	60.13
C18:0 Stearic acid	7.54	2.59	2.39
C18:3 ω-3 α-linolenic acid	2.83	33.88	5.66
ω-3 PUFA	5.25	35.88	6.93
ω-6 PUFA	26.20	32.90	60.22
SFA	58.86	15.64	14.32
MUFA	9.43	15.43	18.44
PUFA	31.45	68.78	67.15
ω-6 PUFA/ω-3 PUFA	4.99	0.92	8.68

Note: SFAs, saturated fatty acids; MUFAs, monounsaturated fatty acids; PUFAs, polyunsaturated fatty acids.

**Table 3:** Effects of dietary linseed oil and sunflower oil supplementation on the growth performance of Hu sheep.

Items	Treatments			SEM	Contrast 1	Contrast 2
	POS	LO	SO		*p*-Value	*p*-Value
Initial body weight (kg)	20.29	20.04	20.19	0.87	0.872	0.908
Final body weight (kg)	43.39	44.38	46.86	1.69	0.290	0.308
Average daily gain (kg/day)	0.28	0.29	0.30	0.22	0.127	0.196

Note: Contrast 1 tested the overall effect of PUFA supplementation (POS vs. LO + SO). Contrast 2 tested the difference between ω-3 (LO) and ω-6 (SO) PUFA sources. Data are based on orthogonal contrast analysis using SPSS. SEM, standard error of the mean.

**Table 4:** Effects of dietary linseed oil and sunflower oil supplementation on serum biochemical parameters in Hu sheep.

Items		Treatment		SEM	Contrast 1	Contrast 2
	POS	LO	SO		*p*-Value	*p*-Value
TP (g/L)	61.45	62.62	64.25	1.15	0.173	0.327
ALB (g/L)	32.27	33.26	31.79	1.14	0.883	0.405
TC (mmol/L)	1.87	1.53	1.58	0.13	0.067	0.771
TG (mmol/L)	0.13	0.14	0.13	0.02	0.846	0.827
CREA (μmol/L)	64.60	56.01	54.16	2.03	0.001	0.524
Urea (mmol/L)	9.57	9.39	8.49	0.50	0.318	0.216
UA (μmol/L)	18.51	16.72	17.21	0.44	0.008	0.438
ALP (U/L)	450.67	481.11	420.95	51.81	0.995	0.419
AST (U/L)	129.44	109.30	97.52	8.17	0.015	0.317
ALT (U/L)	13.64	14.57	13.70	1.41	0.778	0.664
TBIL (μmol/L)	1.40	1.36	0.96	0.21	0.362	0.195
HDL (mmol/L)	0.77	0.53	0.59	0.05	0.003	0.327
LDL (mmol/L)	0.52	0.52	0.52	0.06	0.173	0.405

Note: (1) Serum biochemical indices included total protein (TP), albumin (ALB), total cholesterol (TC), triglyceride (TG), creatinine (CREA), urea, uric acid (UA), alkaline phosphatase (ALP), aspartate aminotransferase (AST), alanine aminotransferase (ALT), total bilirubin (TBIL), high-density lipoprotein cholesterol (HDL), and low-density lipoprotein cholesterol (LDL).

**Table 5:** Effects of dietary linseed oil and sunflower oil supplementation on rumen tissue morphology in Hu sheep.

Items	Treatments			SEM	Contrast 1	Contrast 2
	POS	LO	SO		*p*-Value	*p*-Value
Papilla length/μm	1656.14	1563.41	1382.85	65.58	0.194	0.292
Papilla width/μm	379.50	374.49	362.48	10.69	0.642	0.679
Stratum corneum thickness/μm	20.95	21.46	21.24	0.14	0.192	0.561
Stratum granulosum thickness/μm	19.38	19.00	19.16	0.14	0.312	0.659
Stratum spinosum + basal thickness/μm	63.32	65.46	65.15	0.59	0.120	0.840
Total epithelial thickness/μm	103.65	105.92	105.56	0.60	0.104	0.812

Note: Contrast 1 tested the overall effect of PUFA supplementation (POS vs. LO + SO). Contrast 2 tested the difference between ω-3 (LO) and ω-6 (SO) PUFA sources. No significant differences were observed for rumen tissue morphology parameter (*p* > 0.05 for both contrasts). Data are based on orthogonal contrast analysis using SPSS.

**Table 6:** Effects of dietary linseed oil and sunflower oil supplementation on ruminal VFA profiles in Hu sheep.

VFA Proportion (%)		Treatment		SEM	Contrast 1	Contrast 2
	POS	LO	SO		*p*-Value	*p*-Value
Acetate	58.51	55.27	50.99	1.53	0.008	0.057
Propionate	24.83	21.67	22.97	0.44	<0.001	0.046
Isobutyrate	2.88	4.17	4.47	0.52	0.032	0.681
Butyrate	8.32	10.96	12.31	0.32	<0.001	0.007
Isovalerate	2.54	5.83	6.97	0.45	<0.001	0.085
Valerate	2.92	2.10	2.29	0.39	0.141	0.737
A/P	2.37	2.55	2.22	0.07	0.816	0.003
TVFA (mmol/L)	104.38	144.48	190.58	4.46	<0.001	<0.001

Note: A/P = acetate/propionate. Total VFAs include acetate, propionate, isobutyrate, butyrate, isovalerate, and valerate. Contrast 1 tested the overall effect of PUFA supplementation (POS vs. LO + SO). Contrast 2 tested the difference between ω-3 (LO) and ω-6 (SO) PUFA sources. Data are based on orthogonal contrast analysis using SPSS. Orthogonal contrast analysis was used as the primary statistical method.

**Table 7:** Effects of dietary linseed oil and sunflower oil supplementation on the relative abundance of rumen microbiota in Hu sheep at the phylum level (top 10).

Item	Treatment			SEM	Contrast 1	Contrast 2
	POS	LO	SO	*p*-Value	*p*-Value	*p*-Value
Bacteroidota	59.47	67.02	66.97	2.09	<0.001	0.736
Firmicutes	24.98	26.97	25.63	2.04	<0.001	0.476
Proteobacteria	13.86	1.79	4.53	2.32	0.001	0.475
Euryarchaeota	0.87	1.79	1.4	0.36	<0.001	0.693
Spirochaetota	0.07	1.07	0.39	0.18	0.058	0.980
Fusobacteriota	0.04	0.20	0.07	0.12	0.100	0.362
Verrucomicrobiota	0.04	0.39	0.26	0.12	0.104	0.131
Cyanobacteria	0.01	0.02	0.13	0.07	<0.001	0.845
Actinobacteriota	0.11	0.25	0.19	0.06	<0.001	0.115
Desulfobacterota	0.40	0.21	0.09	0.04	<0.001	<0.001

Note: Contrast 1 tested the overall effect of PUFA supplementation (POS vs. LO + SO). Contrast 2 tested the difference between ω-3 (LO) and ω-6 (SO) PUFA sources. Data are based on orthogonal contrast analysis using SPSS.

**Table 8:** Effects of dietary linseed oil and sunflower oil supplementation on the relative abundance of rumen microbiota in Hu sheep at the genus level (top 10).

Item		Treatment		SEM	Contrast 1	Contrast 2
	POS	LO	SO		*p*-Value	*p*-Value
*Prevotella*_7	38.36	3.13	4.74	3.33	<0.001	0.736
*Prevotella*	5.85	37.79	34.04	3.67	<0.001	0.476
*Succinivibrionaceae*_UCG-001	11.07	0.49	2.66	2.12	0.001	0.475
*Prevotella*_9	8.31	0.17	0.80	1.11	<0.001	0.693
*Syntrophococcus*	1.74	0.18	0.16	0.65	0.056	0.980
*Succiniclasticum*	1.34	2.75	4.05	0.99	0.100	0.362
*Ruminococcus*	1.28	3.17	1.85	0.6	0.104	0.131
*Dialister*	3.98	0.04	0.18	0.5	<0.001	0.845
*Rikenellaceae*_RC9_gut_group	0.31	2.65	3.71	0.46	<0.001	0.115
*Prevotellaceae*_UCG-001	0.93	4.98	2.05	0.46	<0.001	<0.001

Note: Contrast 1 tested the overall effect of PUFA supplementation (POS vs. LO + SO). Contrast 2 tested the difference between ω-3 (LO) and ω-6 (SO) PUFA sources. Data are based on orthogonal contrast analysis using SPSS.

**Table 9:** Alpha-diversity analysis of rumen microbial communities.

Item		Treatment		SEM	Contrast 1	Contrast 2
	POS	LO	SO		*p*-Value	*p*-Value
chao1	363.47	621.06	631.42	35.50	<0.001	0.828
pielou_e	0.61	0.67	0.67	0.02	0.003	0.880
shannon	5.17	6.21	6.19	0.17	<0.001	0.932
Simpson	0.93	0.94	0.95	0.01	0.113	0.506
observed_features	344.90	600.00	607.40	34.33	<0.001	0.878

Note: Contrast 1 tested the overall effect of PUFA supplementation (POS vs. LO + SO). Contrast 2 tested the difference between ω-3 (LO) and ω-6 (SO) PUFA sources. Data are based on orthogonal contrast analysis using SPSS.

**Table 10:** Analysis of differences in rumen microbial community structure among treatment groups.

	PERMANOVA			ANOSIM	
	F	R^2^	*p*-Value	R	*p*-Value
POS vs. LO	13.72	0.433	0.001	0.937	0.001
POS vs. SO	10.86	0.376	0.001	0.788	0.001
LO vs. SO	1.40	0.072	0.080	0.115	0.052

## Data Availability

The datasets generated and/or analyzed during the current study are available from the corresponding author upon reasonable request.
